# Pericardioperitoneal shunt for the treatment of refractory non-malignant pericardial effusion

**DOI:** 10.1093/icvts/ivac215

**Published:** 2022-08-22

**Authors:** Ishida Masaru, Kajiyama Tetsuya, Satoh Hisashi

**Affiliations:** Department of Cardiovascular Surgery, Higashi Takarazuka Satoh Hospital, Takarazuka, Japan; Department of Cardiovascular Surgery, Higashi Takarazuka Satoh Hospital, Takarazuka, Japan; Department of Cardiovascular Surgery, Higashi Takarazuka Satoh Hospital, Takarazuka, Japan

**Keywords:** Pericardioperitoneal shunt, Denver shunt, Pericardial effusion

## Abstract

We report the case of a 74-year-old man treated for refractory non-malignant pericardial effusion using a pericardioperitoneal shunt. After the failure of conventional pericardiocentesis, a pericardioperitoneal shunt using a Denver shunt was inserted to drain the pericardial effusion into the peritoneal cavity. At 3-year follow-up, the effusion was well controlled and the shunt remained patent.

## INTRODUCTION

Pericardial effusion is a common finding in clinical practice, whether incidental or as a manifestation of a systemic or cardiac disease [1]. Pericardiocentesis should be considered when the pericardial effusion becomes symptomatic or when empiric anti-inflammatory medication is unsuccessful. However, reaccumulation of pericardial effusion often occurs within a few weeks of the procedure. Although various surgical approaches for pericardial window creation for drainage have been proposed, these radical procedures remain controversial. We report a case of long-term pericardioperitoneal shunt placement as a less-invasive alternative treatment for refractory non-malignant pericardial effusion.

## CASE REPORT

**Figure 1:**
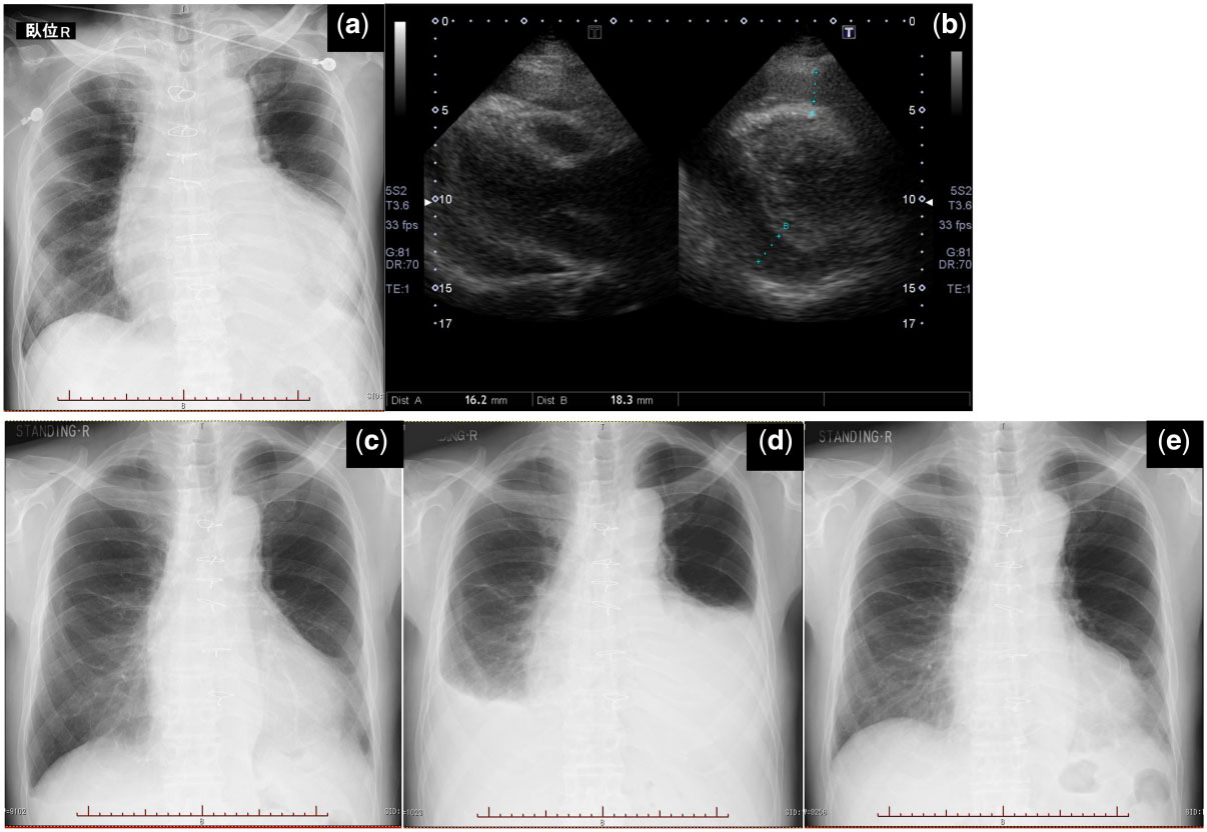
Chest radiography and echocardiography on initial admission (**a** and **b**), at discharge (**c**), on readmission (**d**) and at 3-year follow-up (**e**).

**Figure 2:**
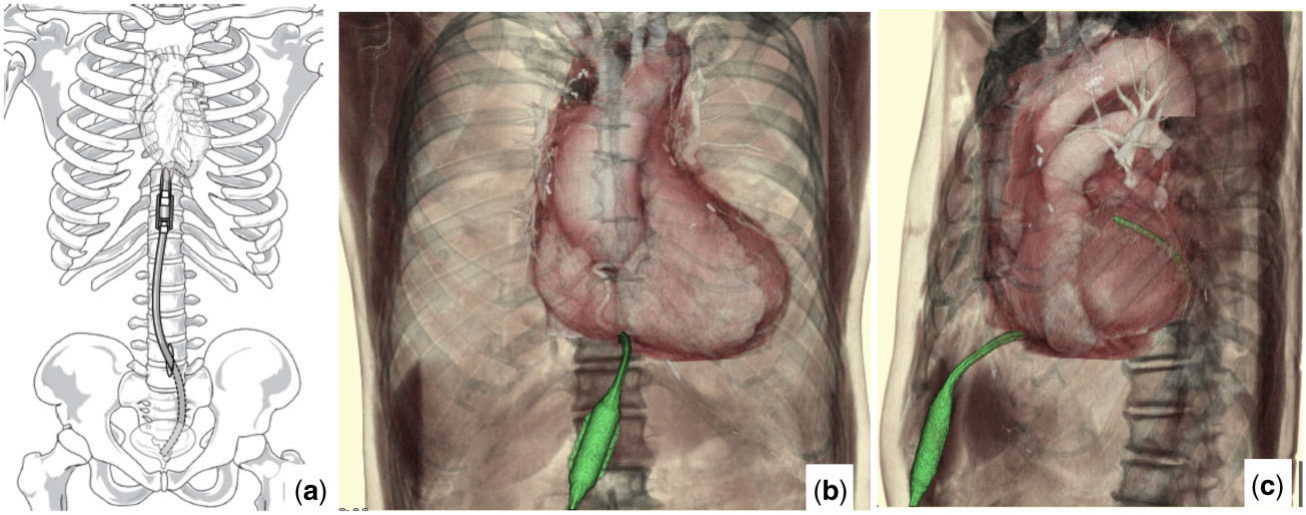
Placement of a pericardioperitoneal shunt. Illustration of shunt positioning (**a**). The inflow end of the shunt was placed in the pericardial space, whereas the abdominal end was tunnelled subcutaneously and inserted into the peritoneal cavity. The computed tomography images of the shunt (green): frontal (**b**) and left-side (**c**) views (A color version of this figure appears in the online version of this article).

A 74-year-old man was transferred to our emergency department with spontaneous recovery from temporary loss of consciousness. This patient underwent resection of a well-differentiated mediastinal liposarcoma with adjuvant radiation therapy when he was 71 years old. Although there was no tumour recurrence, mild pericardial effusion was diagnosed at the age of 72 years. Upon admission, he was restless and haemodynamically unstable, with a blood pressure of 64/53 mmHg, a heart rate of 120 bpm and an increased respiratory rate. Physical examination revealed cold, sweaty extremities. Electrocardiography revealed sinus tachycardia with low QRS voltage. Chest radiography revealed markedly enlarged cardiac silhouette (Fig. 1a). Troponin T was negative and brain natriuretic peptide was 159 pg/ml. Echocardiography revealed a large pericardial effusion with cardiac tamponade physiology (Fig. 1b). The patient underwent emergent pericardiocentesis with echocardiography guidance. Approximately 260 ml of bloody pericardial effusion was evacuated, and an aspiration silicone tube was placed into the posterior pericardial recess. The patient experienced immediate improvement in haemodynamic status. Pericardial effusion analysis was negative for infection. Cytology showed no malignant cells, and acid-fast bacterial staining was negative. Two weeks after the procedure, chest radiography revealed improved cardiac silhouette (Fig. 1c), and the patient was discharged. However, he returned to our hospital complaining of dyspnoea 36 days after discharge, with a large reaccumulation of the pericardial effusion (Fig. 1d). Treatment with a pericardioperitoneal shunt using a Denver shunt (Mihara Medical, Inc., Tokyo, Japan) was performed (Fig. 2). With the patient supine under general anaesthesia, a 10-cm incision was made over the xiphoid, and subxiphoid pericardiotomy was performed. A small subcutaneous pocket was created through the subxiphoid incision to accommodate the pump apparatus. The inflow end of the shunt was placed in the pericardial space. The abdominal end of the shunt was tunnelled subcutaneously and inserted via a 3-cm midline incision into the peritoneal cavity in the lower abdomen. The pump was manually compressed twice a day. At 3-year follow-up, the patient was asymptomatic (Fig. 1e). Echocardiography and abdominal ultrasound showed no pericardial effusion or ascites.

## DISCUSSION

A wide variety of conditions can result in pericardial effusion. Common causes include cancer (10–25%), pericarditis and infection (15–30%), iatrogenic (15–20%) and connective tissue disease (5–15%), while up to 50% are idiopathic [2]. In our patient, inflammatory disease and neoplasm were ruled out by pericardial fluid examinations. White blood cell count and C-reactive protein levels were normal. Laboratory tests excluded connective tissue disease. We were unable to definitively prove the cause of the pericardial effusion. A large, chronic, idiopathic pericardial effusion, defined as pericardial effusion that persists for >3 months with no apparent cause, has a risk of progression to cardiac tamponade. Pericardiocentesis and tube drainage are the first steps in the diagnosis and treatment of right heart collapse. Although the safety of pericardiocentesis with echocardiography guidance has improved, pericardiocentesis alone is associated with reaccumulation of pericardial effusion [3]. Surgical pericardiotomy or less-invasive options should be considered. The use of a Denver pleuroperitoneal shunt has been previously described for the treatment of refractory pleural effusion [4]. Wang *et al.* [5] introduced pericardioperitoneal shunting with a Denver shunt to treat malignant pericardial effusion. The device was used to treat 4 patients with the aim of palliation only; the median survival of the patients was 3.1 months. It is important to assess long-term durability and freedom from complications, including device thrombosis and infection, in patients with non-malignant pericardial effusion. Our patient has been free from cardiac symptoms and device complications for 3 years. Under the close monitoring, 6-month follow-up echocardiography revealed no significant pericardial effusion. Fusion of the pericardium to the epicardium is expected because pericardial effusion is minimal. However, because there was no evidence of inflammatory fusion, the shunt system was maintained and the patient performed pump compression daily. All outpatient physical examinations and 6-month echocardiography showed no infectious complications.

In conclusion, pericardioperitoneal shunting using a Denver shunt is a less invasive but equally effective alternative treatment for chronic non-malignant pericardial effusion.

**Conflict of interest:** none declared.
